# Development, implementation and evaluation of the digital transformation of renal services in Wales: the journey from local to national

**DOI:** 10.1007/s11096-022-01466-9

**Published:** 2022-10-28

**Authors:** E. Mantzourani, O. Brooks, D. James, A. Richards, K. Hodson, H. Akhtar, M. Wakelyn, L. White, R. Williams, G. O’Gorman, A. Kervin, J. Chess, C. Brown

**Affiliations:** 1 Cardiff School of Pharmacy and Pharmaceutical Science, Cardiff University, Cardiff, Wales UK; 2 Renal Pharmacy, Swansea Bay University Health Board, Swansea, Wales UK

**Keywords:** Electronic prescribing patient care, Kidney disease, Patient portal, Quality of care

## Abstract

**Background:**

Treatment for people with kidney disease is often associated with complicated combinations of medicines. Logistical challenges with traditiona paper-based prescribing means that these patients are particularly susceptible to medication-relation errors and harm.

**Aim:**

To improve the quality of care that people with kidney disease receive across Wales through a Value-Based digital transformation programme.

**Setting:**

Renal units within the National Welsh Renal Clinical Network (WRCN).

**Development:**

A novel Electronic Prescribing & Medicines Administration (EPMA) system, integrated into a patient care record and linked to a patient portal was developed in South West Wales (SWW) region of the WRCN, enabled by the Welsh Government (WG) Efficiency Through Technology Fund. National upscale was enabled through the WG Transformation Fund.

**Implementation:**

EPMA was designed and rolled out initially in SWW region of the WRCN (2018). A dedicated delivery team used the blueprint to finalise and implement a strategy for successful national roll-out eventually across all Wales (completed 2021).

**Evaluation:**

A multi-factorial approach was employed, as both the technology itself and the healthcare system within which it would be introduced, were complex. Continuous cycles of action research involving informal and formal qualitative interviews with service-users ensured that EPMA was accessible and optimally engaging to all target stakeholders (patients and staff). Results confirmed that EPMA was successful in improving the quality of care that people with kidney disease receive across Wales, contributed to Value-Based outcomes, and put people who deliver and access care at the heart of transformation.

**Conclusion:**

Key findings of this study align directly with the national design principles to drive change and transformation, put forward by the WG in their plan for Health and Social Care: prevention and early intervention; safety; independence; voice; seamless care.

## Facilitators of best practice

- Development of an in-house product, tailored to suit the needs of the service, accompanied by a suite of digitally accessible, lean and paper-lite training materials to support flexible learning.
- Co-creation with service-users (patients and staff) and engagement of key stakeholders at all stages.
- Clear implementation strategy, incorporating a project initiation period, with a dedicated team of clinical staff, experienced in technology.

## Barriers to best practice

- Time for staff to be released for training—overcome by:
  - lean training material in the form of short videos;
  - real time one-to-one training in the care setting.
- Across Health Board information technology (IT) barriers—overcome by a dedicated renal IT engineer to coordinate roll-out with local IT teams.
- Limited engagement from some key staff as a results of work pressures—overcome by:
  - identifying local enthusiasts and agile use of local skill mix;
  - have a multi-professional central Delivery Team agile to fill local gaps in skill mix.

## Background

In 2018, following a Parliamentary review, the Welsh Government (WG) published their long-term plan for Health and Social Care [1]. ‘A Healthier Wales’ described the need for change in order to meet the needs of the Welsh population. It set out a vision for a ‘whole system approach’, integrating health and social care, with an emphasis on using new technologies to support people in managing their own health and wellbeing, and to enable them to live independently.

In Wales, renal services are coordinated from three regional centres in the South West, South East and North of the country. The Welsh Renal Clinical Network (WRCN) commission and plan specialised renal services. With a growing elderly and comorbid population the demand for specialised renal services is increasing. Current estimation in Wales is that around 6% of the adult population has chronic kidney disease (stages 3–5), when renal services are required to support the specialist treatments and care of the patient [2].

The South West Wales Renal Service (SWW-RS) cares for people with kidney disease over a large geographical area, including people with progressive kidney disease, kidney failure, and those who require dialysis or kidney transplantation. For these patients, treatment is often associated with a complicated combination of medicines that are prescribed and monitored by specialist prescribing pharmacists, prescribing nurses, and renal physicians. The supply is done by renal pharmacy professionals [3]. SWW-RS has a dedicated renal pharmacy, which is professionally-integrated into the regional multi-professional service. This has created the culture to think differently to redesign services, to provide equitable, accessible, and high-value treatments and care to people with kidney disease [4].

Logistical challenges with traditional paper based prescribing in the regional SWW service, which covers a large geography with a number of rural satellite dialysis units, often resulted in treatment delays, missed medication doses and mismatches between paper prescriptions and electronic records, as reported elsewhere [5] with patient safety compromised. In addition, clinicians wasted time on administrative tasks and unnecessary travel and relied on fax machines.

It became evident that delivering value and capacity to deal with the growing pressures in the healthcare system, whilst also aligning with the World Health Organisation’s (WHO) Global Patient Safety Challenge: *Medication Without Harm* [6], meant doing things differently.

### Aim

To improve the quality of care that people with kidney disease receive across Wales through a Value-Based digital transformation programme, in line with WG and WHO strategy.

## Development

As part of a national transformation programme, kidney care in Wales has been digitised through a single platform: the renal electronic patient record (rEPR). rEPR is a single platform for information sharing, allowing patients (via a patient portal) and healthcare professionals to easily access information online. The purpose of this digitisation was to improve the quality of care for all patients who access renal care services in the country and support them with self-care [7]. SWW-RS identified the need to further digitise their service and develop a new Electronic Prescribing and Medicines Administration (EPMA) module within their rEPR.

EPMA computerised systems have been designed to address some of the problems associated with paper-based drug charts, and have the capacity to facilitate both the prescribing for patients and the subsequent administration of these medicines within hospitals [5]. Several studies have built the evidence-base supporting the positive clinical use of these systems, with results confirming reductions in prescribing and administration error rates [8], reduced illegibility issues and hence decreased likelihood of medication errors [9]. In addition, they demonstrated other benefits such as cost-savings to the NHS, staff effectiveness and workflow, accountability and auditing, formulary compliance and out-of-hours support, freeing up clinicians’ time from duplicating information in more than one records [10]. Routinely collected data of EPMA systems have also been used to generate actionable knowledge to improve medication safety [11].

However, EPMA systems do not come without challenges and realising the benefits that digitisation can bring is contingent to addressing several considerations. It has been argued that EPMA can disrupt workflows and work patterns, and have a negative effect on communication between members of the multidisciplinary team [12–15]. In some cases, no impact on medication errors has been found and even an increase in discrepancies has been reported [16]. Issues such as incorrect drug picking, population of dosages and remote prescribing have been identified as contributing to these errors [17].

Despite this, EPMA is established in many NHS Trusts in England and in other countries. Unlike many of these hospitals, SWW renal EPMA is fully integrated with the renal central electronic patient record. The team were conscious that digitisation should be more than simply implementing new technology, it should reconceptualise the service delivery model and must put people who deliver and access care at the heart of transformation. Hence, the development, implementation and evaluation of EPMA was completed in phases, informed at all stages by the updated Medical Research Council framework for the development and testing of complex interventions [18]. This included identifying the desired digital approach, developing it, assessing its feasibility ensuring we noted uncertainties and refining points, continuous evaluation and implementation. It was a dynamic process involving an adaptive design, whereby we aimed to explore how the system works, what the direct and indirect impact was and how it impacts on system change, and what the balance of the resources versus outcomes was. It was decided that a ‘retrofitted’ commercial EPMA product would not be the solution; instead a fit-for-purpose product was designed in-house (insight from nurses available at: https://youtu.be/vOTRuurgwgA): it was simple to use but elegant to deal with complexities of renal treatment, affordable, integrated within the existing rEPR rather than overcoming interoperability issues, with the team having control over its evolution based on feedback. The product also allowed all users the professional autonomy to undertake auditable clinical decisions when at patient’s best interest, and the professional decision to act was not prohibited by a ‘computer says no’ permission or functionality issue.

The pharmacy team were involved in all parts of the process from securing funds and budgetary responsibility and appropriate approvals, through to concept design and technical development, providing training materials and face to face training, ongoing support and ongoing development. The digital intervention was co-designed with service-users in an iterative approach (core development team of four renal pharmacists, one pharmacy technician, one nephrologist, one IT technician, one project manager and one nurse in the SWW-RS), using multiple cycles of action research [19, 20] and following principles of the person-based approach to development of digital health-related behaviour change interventions [21]. This ensured EPMA was accessible and optimally engaging to all target stakeholders, including a wide range of healthcare staff (pharmacists, nurses, dietitians, physicians etc.) and patients, and that promoting change and problem-solving was embedded throughout the whole journey. At the heart of the strategy was to make information available to patients via the digital patient portal, or to automate the outputs to paper, for patients who preferred to assess their information in this way. Patients were supported to understand and act on this information to be active partners in managing their health.

**Fig. 1:**
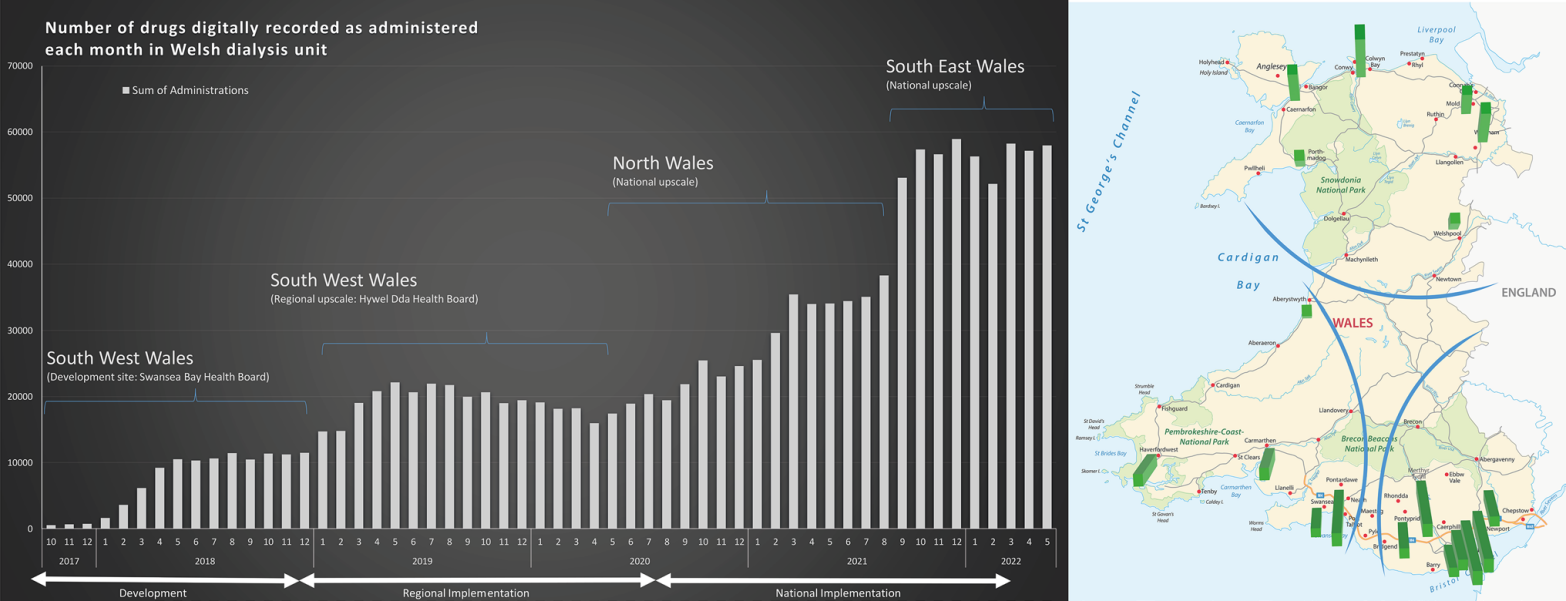
Number of medication digitally recorded as administered each month in Welsh dialysis units since start of development of EPMA, noting the start and end of each respective development and/or roll-out period (*left*) and the location of the 18 dialysis units across the three regions in Wales (*right*). The height of each rectangle represents the size of the respective unit, in terms of patient population numbers

Early cycles of action research in the development phase involved qualitative think-aloud techniques [22] and verbatim extracting positive and negative comments about the system, observing and noting reactions to the different functionalities. The bespoke EPMA module was rolled-out to five dialysis units across SWW by 2019 (Fig. 1), providing equitable care for people living in rural and urban areas (development journey available at: https://youtu.be/L2KaMC41RIg). In this stage, cycles of action research involved formal interviews with staff and patients to supplement the initial, informal feedback. The approach facilitated informed discussions and constant improvement, with documented proposed changes, their rationale and priority [23]. It also allowed identification of enablers for the wider roll-out, from one dialysis unit in one hospital, to the whole of Wales. Some of the evidenced-based adaptations, tailored to service-users, were the training model, including short videos for training of nursing staff rather than relying on a manual; continuing co-creation with service-users to make the system intuitive and improve user-friendliness; improved reports on medication use and simple business continuity procedures in case of system failure. Collaboration with the commercial partner chosen to develop the system and WG funds (Efficiency Through Technology Fund (2016), Transformation Fund (2020)) facilitated both the system development and the service transformation.

The core EPMA development principles were:

- a paper-free tool where treatments prescribed from any renal centre are instantly available for a patient wherever they are receiving care;
- system housed within the established single rEPR;
- bespoke touch-screen medicines module designed in-house by the end users;
- integration with the patient portal;
- incorporating business and clinical intelligence in real time, including medicines surveillance dashboards.

## Implementation

Having created the blueprint with the successful regional implementation, a strategy was developed for national implementation. Government funds allowed for the original SWW development team to be designated as a Transformational scale-up Delivery Team, consisting of renal health professionals, renal IT engineer and a newly enrolled programme manager who engaged with the commissioner and renal colleagues across Wales. This experienced team enabled the successful national roll-out at scale, at pace and within budget. Non-staff costs included hardware and software development, and external evaluation.

The EPMA module was designed with scalability and flexibility in mind, to accommodate warranted variation in practice across the regions; however, a clear implementation strategy for roll-out was essential. EPMA was implemented in 13 dialysis units across the two remaining Welsh renal regions between 2020 and 2021. The core delivery team introduced EPMA in each unit and outlined the process of change with renal physicians, nurses and pharmacists as described above in advance of each unit’s ‘go-live’ date. Media, such as videos and infographics, were used for engagement to tell the story of the project and transformation; these resources were made accessible to those unable to attend events.

Digital hardware, including touchscreen computers and combination medication and computer carts were purchased by the Delivery Team and provided to each dialysis unit to standardise the model across the country. The team worked with local IT departments in each locality (Health Board) to prepare the dialysis unit for EPMA, ensuring good WiFi connectivity and robust business continuity procedures in case of system or network failure.

Each dialysis unit was assigned with up to one week with onsite support from the Delivery Team to transition from paper-based prescribing to paper-free EPMA. Pharmacy professionals, an IT engineer and project manager from the Delivery Team travelled to each dialysis unit to support local clinicians. This included the transcribing of paper-based medication records onto the new electronic module, followed by digital authorisation by the unit’s prescribing pharmacists or physicians. Prescribers and nurses were provided with face-to-face training and competency was assessed by parallel running paper medicines charts with the replicated e-chart. The core delivery team provided ongoing clinical and technical support remotely and provided virtual pharmacy surveillance (using real-time clinical intelligence reports) to each dialysis unit after EPMA implementation, to ensure quality and patient safety.

## Evaluation

Evaluating EPMA required a multi-factorial approach, as both the technology itself, as well as the healthcare system within which it would be introduced, were complex [24, 25]. Table 1 outlines the on-going projects that go beyond the question of whether the intervention works; as outlined by Skivington et al.[18], a broader set of questions is being asked related to understanding the impact of the system on different health and social care outcomes, and the financial implications, as well as the preliminary findings. In this section the detailed findings of interviews with patients will be presented. It is important to note that multiple team meetings have already taken place to triangulate results from the different parallel projects, using a formalised protocol [26].

**Table 1:** Overview of the different strands of the multi-factorial approach to evaluating the renal digital system in Wales

Evaluation strand	What has been/is being done	What we have found
Time spent on patient care Context: Medication (erythropoiesis stimulating agents and intravenous iron) for renal anaemia are administered on each dialysis session. Each month treatment is reviewed to maintain tight treatments targets Changes or additions to medication that are administered on dialysis can now be made from any location and will be immediately accessible to the nurse at any satellite unit. The monthly review of blood results needed to monitor treatments can be centralised, or localised, depending on need and clinician availability Clinicians no longer need to travel for the purpose of making administrative changes to paper records	EPMA, available at any renal unit, has enable virtually and centralised review—with digital clinical surveillance tools assisting target intervention Time in motion study: South West Wales renal anaemia service Case study: Nephrologist clinic review of satellite haemodialysis unit in South West Wales (approximately 2 h drive from main centre) With paper based prescribing this allocation was spent on administrative tasks of re-writing drug charts and prescribing, meaning little face to face time with the patient	Liberation of significant clinical and administrative time: Extrapolation from an evaluation in one region: EPMA has released the equivalent of 14 clinical sessions regionally or 42 clinical sessions across Wales each month NB: clinical session defined as half day Reduction in travel time and costs for clinic staff: In one region’s satellite unit, four days per month were committed to visiting three satellite units to prescribe anaemia treatments. This is now achieved in one day with no travelling In the main unit, five days per months were committed to prescribing treatments at the three main units. This now achieved in three days Improving timeliness of patient care: Intended changes to medications (decided after monthly blood tests) would be delayed by 2 or 10 days for patients based in remote satellite units, depending on next scheduled visit of prescriber (unless urgent where fax was utilised)
Staff perceptions of new system	Nineteen interviews completed with healthcare staff of different roles (lead nurse, nurse, pharmacist independent prescribers (IP), pharmacist non-IPs, dietician, consultant) Deductive analysis: Completed prior to the digital system rolling out to units outside of the South West Wales region of the Welsh Renal Clinical Network, to identify enablers for wider roll-out Framework analysis: Against the Institute of Medicine’s six domains of healthcare quality	Suggestions for improvements were continuously fed back to the team, to enable timely action before further roll-out Participants’ responses have been linked to all of the healthcare quality domains, highlighting the holistic impact of the digital system
Patient quality and safety Context: In 2019: 5,000 drugs per week administered on haemodialysis in SWW	Rates of missed doses (or unrecorded administrations) calculated for drugs administered on unit haemodialysis Pre EPMA: All paper chart audit in main dialysis unit during March 2017. Sample n = 6390 scheduled medication administrations Post EPMA: Automatic medicines surveillance report in all dialysis units during one week in March 2019. Sample n = 5033 scheduled medication administrations	A 96% reduction in missed doses was estimated: Pre EPMA: 3.7% (185 missed / 5000 scheduled doses) Post EPMA: 0.1% (7 missed / 5000 scheduled doses)
Discrepancies in care records Context: Prior to EPMA, electronic care record served solely as a repository of medicines for the patient, not as a prescription. In theory it should have reflected the paper medication chart EPMA is now the single prescribing and administration record for medication; concurrent paper record no longer used	Calculated rate of discrepancies between paper and electronic records pre EPMA, in one satellite unit in SWW, during February 2019. Data collected during manual transcription of paper charts at the point of rollout of EPMA to the unit. Sample n = 177 prescribed drugs, 45 patients	Pre EPMA: 19% of prescribed drugs did not match the renal Electronic Patient Record 14% of prescribed drugs were not recorded on the renal Electronic Patient Record 22% of renal Electronic Patient Records did not match the allergy status on the paper chart Post EPMA: zero discrepancies

### Study design, method and data analysis

As part of the wider project consisting of action research cycles to evaluate the staged national digital transformation across renal units in Wales, we wanted to explore patients’ experiences of treatment in the renal units using the new EPMA system and how the system has impacted patient experiences and outcomes. A qualitative phenomenological design was used for exploring patient views, employing semi-structured interviews. Further details on design and ethical considerations are provided in Table 2.

**Table 2:** Details on the data collection, sample and recruitment (including inclusion/exclusion criteria), sample size, sampling technique, recruitment and consent, and ethical and regulatory considerations for the patient evaluation strand of the study

Methodological aspect	Detailed information
Data collection	Interviews were conducted by the researcher over the phone or through Skype/Teams with or without the use of video (whatever was preferred by each participant, guided by a semi-structured interview schedule and lasting approximately 30–40 min. Written consent was sought from participants prior to the start of the interview. The interviews were audio or video recorded for transcription, with consent, and once verbatim transcription was complete the recordings were deleted to ensure data anonymisation
Sample and recruitment	Patient/guardian/carer
*Inclusion criteria*	
Population	Individuals or carers of individuals who have been initiated or on maintenance HD therapy, or require treatment for chronic kidney disease, since the introduction of the EPMA system Over the age of 16
Experiences	Individuals who have been initiated or on maintenance HD therapy since the introduction of the EPMA system
Communication	Can communicate effectively in English (does not have to be first language), to be assumed upon response to the information leaflet Ability to provide informed consent for themselves or the patient they care for
*Exclusion criteria*	
Population	Individuals or carers of individuals who require HD treatment or treatment for chronic kidney disease, and access this through a renal unit in Wales without the EPMA system Under 16 years of age
Communication	Unable to provide informed consent
Sample size	Using the principles of information power, it was estimated that between 7–15 participants from all patient populations would be needed to gain valuable data across the whole SWW region, to account for a representative sample
Sampling technique	Potential participants were selected using a mix of non-probability purposive and convenience sampling a. Purposive: Potential participants were screened against the eligibility criteria presented above and approached by the gatekeepers b. Convenience: Participants were self-selected, as they chose to respond to the information leaflet
Recruitment and consent	Designated members of staff acted as gatekeepers, identified patients that met the inclusion criteria, and provided them with a copy of the information sheet and consent form about the study (in English and Welsh) with the research team’s contact details, so they could contact the researcher directly if they were interested to find out more about the study. In the case of children, patients with difficulties in communicating or who lacked capacity to give informed consent, their carer would be approached. All the basic elements of the consent process were followed and no incentives were offered for participation
Ethical and regulatory considerations	Participation was voluntary, and all participants were informed of their right to withdraw at any point. The study was carried out in accordance with the Research Integrity and Governance Code of Practice, laid out by the NHS and Cardiff University; the project was registered as a service evaluation with NHS Research and Development department, and ethical aspects were assessed by Cardiff University, who granted a favourable opinion (ref: 2021–01). All participant information and characteristics were anonymised, de-identified and given pseudonyms in the final report, all audio or video recordings of interviews were deleted immediately after transcription. At this point there was no way to connect consent forms with individual comments ensuring complete anonymisation of data

Following transcription and familiarisation with the interviews, transcripts were coded and mapped against a combination of two different frameworks, to find out the impact that the digital system has had on patients’ quality of care and their wider lives more generally:

1. The three dimensions of health, as stated in the definition of health by WHO: “…a state of complete physical, mental and social well-being” [27].
2. The six domains of healthcare quality, by the Institute of Medicine [28].

To ensure no other concepts have arisen during the interviews that have not been captured with the framework analysis, inductive analysis followed the approach outlined above.

## Results

Eleven patients were recruited and interviewed between 23^rd^ April 2021 and 30^th^ November 2021 (Table 3).

**Table 3:** Summary of participant characteristics; disease status for all patients was kidney failure. PatientView is the renal patient digital portal [19]

Participant	Age bracket	Sex	Occupation/ former occupation	Length of renal condition (years)	Current renal treatment
Patient Participant (PP) 1	50–60	F	Education	51	Kidney transplant recipient
PP 2	30–40	F	Unknown	22	Home nocturnal (overnight) haemodialysis
PP 3	60–70	M	Engineering	45	Hospital Haemodialysis
PP 4	50–60	M	Engineering	5	Hospital Haemodialysis
PP 5	50–60	F	Healthcare	25	Home nocturnal (overnight) haemodialysis
PP 6	40–50	M	Healthcare	17	Kidney transplant recipient
PP 7	50–60	F	Unknown	5	Home haemodialysis
PP 8	60–70	M	Self-employed	20	Hospital haemodialysis
PP 9	40–45	F	Unknown	20	Kidney transplant recipient
PP 10	60–70	M	Former engineering	4	Home nocturnal (overnight) haemodialysis
PP 11	20–30	M	Healthcare	9	Kidney transplant recipient

**Fig. 2:**
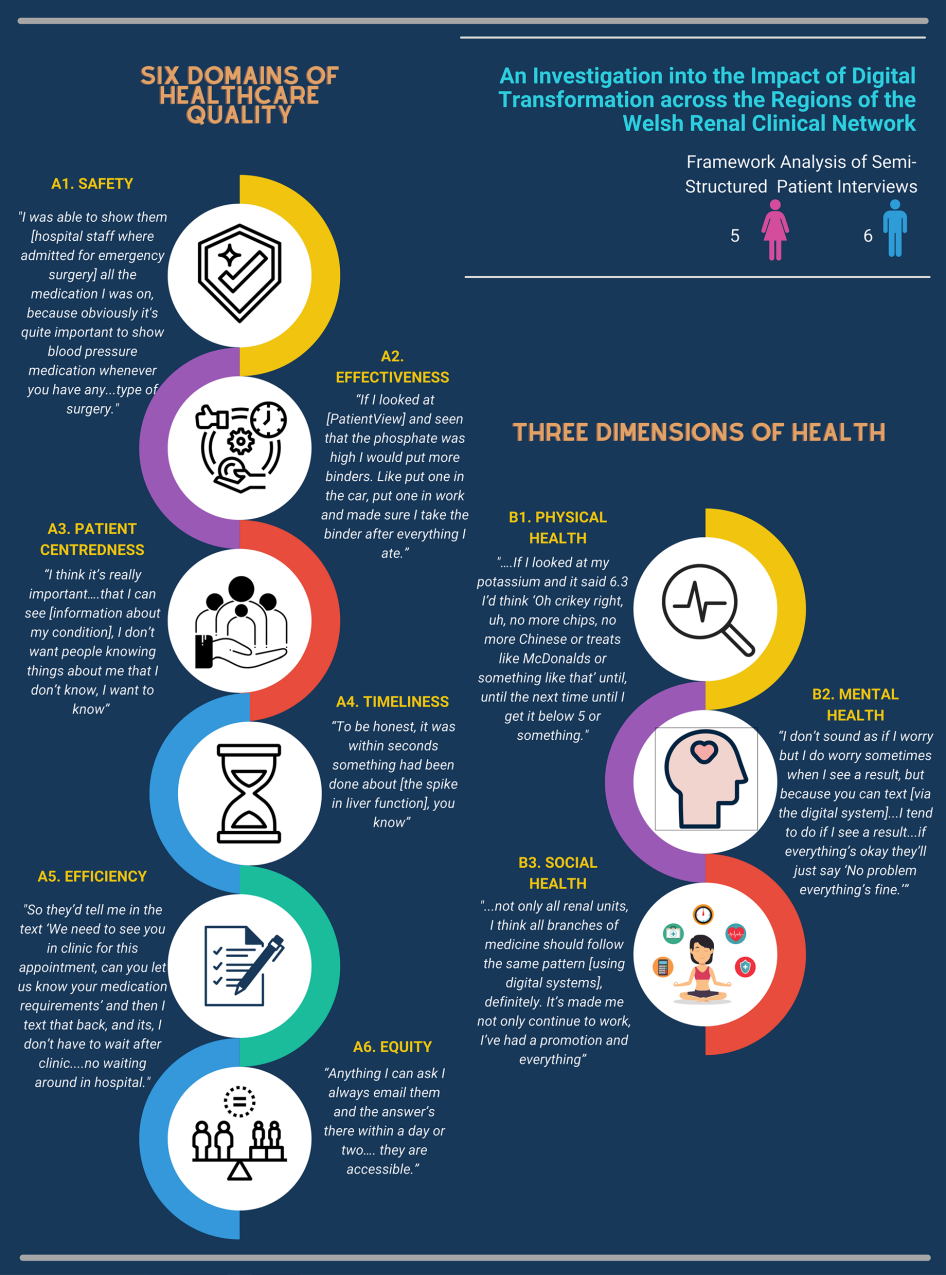
Key quotes from framework analysis of interviews with patients, mapped against the three dimensions of health [27] and the six domains of healthcare quality [28]. Figure created using Canva Education edition

The combined framework, consisting of the six domains of health care quality and the three dimensions of health, provided nine themes for analysis—data was mapped across all domains, highlighting the holistic impact of the digital system on patients (Fig. 2, Table 4). No additional themes were found using inductive analysis.

**Table 4:** A selection of additional quotes that exemplify the findings for each theme, after framework analysis of transcribed patient interviews. All quotes have been anonymised and are identified only by the pseudonym of the participant who said it, for example PP1 for patient participant 1

Domain	Summary of findings and participant quotes
A1: Safety	PP3: “I liked the fact that everyone can see…whether it’s the doctor, the pharmacist, the anaemia nurse….. they can see the same thing and nothing’s hidden.” PP9: “I had to go [to A&E]. I had a contusion in my head. I had a surfboard hitting my head, so I had to say all my tablets…[different time I have] been for tummy ache as well…It was quicker for me to get [blood results on Patient View] than for them to get it on the system.” PP1: “I kept on asking my GP for a specific [drug]… that I was comfortable taking…My GP decided he was going to change it….and when I had the generic one I had a reaction to it……swelling and things……..It’s that type of thing that they can see and they know my history looking on screen [PatientView. Because I’ve got all of the information on my screen as well you know.”
A2: Effectiveness	PP7: “Once the bloods have been done, the next morning I go on to PatientView to check my iron, my haemoglobin, my INR. ….Because if it’s not right then I, I know I can get in touch with [the pharmacist] and we will chat about do I need iron?.” PP2: “Every time I have my blood taken I check PatientView to see what the results are. And then about a week or 10 days later there will be medication changes put up on PatientView, so I’ll have a look at those as well.” PP3: “I go and look at my blood results, especially my monthly blood results, and I look at the trends there.”
A3: Patient centredness	PP1: “I rely on it…..as soon as the message comes and sometimes it comes late at night, I will look at it whatever time…You know, it’s important to me, perhaps it isn’t to other people but to me it’s the way I live now.” PP3: “I can look at the trends going back years and I notice how, for instance, how GFR has deteriorated over the years. Which is a bit of an interest…for me.” PP1: “Because I don’t like…..blood infusions because I’ve always got a funny taste in my mouth…..I get a little bit fed up, when the nurse is by your side the iPad computer [means] she doesn’t have to leave me, you know….there’s always somebody on hand….I’m comfortable going there.”
A4: Timeliness	PP1: “If there’s a problem I’ve got instant access to people…to a text…to emails, you know, there’s always something I can do.” PP1: “When they looked at my blood results there were other minerals and other vitamins I was deficient in, so they prescribed Remevit for me…..they were able to prescribe that there and then for me, you know.” PP11: “When I was with [a different renal unit outside of Wales], I'd have to wait…up to a couple days for some results to be, um, actually looked at by a consultant…Whereas with PatientView, I can view them in the evening and if there is any issues that I see, personally I can phone up that night or the next day and just, uh, go over my concerns.”
A5: Efficiency	PP1: “I like a text I do or otherwise I forget.” PP2: “For me the phone app it works well. I think it helps to cut down on paper as well so I’d rather that over a letter.” PP3: “I’d rather online. It’s quicker, more direct so I’m not waiting for a letter so online is great.”
A6: Equity	PP5: “Oh I find it great because every morning when I come off dialysis I put all my details, my treatment on PatientView…..I just go into….dialysis treatment and then just put all my blood pressures, how much I’ve taken off the machine…the pulse.” PP1: “If I’ve got a question which I have had a few times, for example I’ll just text ‘my potassium is high is that okay?’….and they always reassure me.”
B1: Physical health	PP3: “There’s a menu sheet…so if I’ve come across some food that I’m not sure that is high in protein or high in potassium or whatever I can got to that and that’s a reference point for me.” PP9: “If I see my creatinine is high, but I mean I'm really good with my water so I don't need really to, to be careful, but obviously if it's come a bit high up, then I will make a bit more effort, like I will be conscious, you know, and, and do it a bit more.”
B2: Mental health	PP9: “I know I'm going to have the result between 9:00 and 11:00 and I, I just wait for that and then…I can be relieved or I won't be worried because I know there is always a solution. Um, so it really, really helped me to say ‘Oh brilliant, I'm good’.” PP9: “I love it. I absolutely love it. Um, I don't think I can do without it…it makes me feel really, really good to know my blood results after a blood test.”
B3: Social health	PP6: “I was working for a private ambulance service….I did show them my blood results [on PatientView], saying “look I’m fine, my bloods are normal.” PP3: “If we’re going out for a meal we look at the menu and I can always access it to see, you know, if there’s a possible problem with me eating a certain food.” PP9: “When we apply to, to be part of the race, basically [organisers] want to know all the medicine and…what my condition and what they can [do].” PP4: “You go on PatientView and just click on medication and everything that you’re on is on there….I went on holiday and they asked me if you’re on any medication and I went on there and printed it out.”

### Safety

Patients believed that the digital system had made care safer by allowing healthcare professionals to view the same, up-to-date information. They particularly highlighted how using Patient View, the patient-portal interface of the system, helped ensure that any treatment patients received in non-renal settings would not cause any harm. Examples were provided of cases where circumstances prevented patients from sharing results on PatientView [29], leading to safe transfer of care being compromised.

### Effectiveness

The patient portal was raised as an extremely effective resource in supplementing patients’ care, which allowed patients to actively manage their own condition by checking blood test results and medication changes live, on their phone, and also to input their own data, such as blood pressure and volumes of dialysis fluid after home dialysis. They felt this was effective in enabling them to self-care and be independent.

### Patient centredness

The opportunity that patient portal offered to patients to access their own information was considered important, empowering them to feel involved and at the centre of their care, increasing their awareness of their condition and their treatment. They felt in control as they could monitor trends in their blood results to see whether their condition was improving or deteriorating, and used the parameters that were given to know what to aim for. Patients also gave examples of how the digital system being used by staff had allowed their preferences and needs to be taken in to account, and how it increased bedside interactions, which was welcomed and appreciated.

### Timeliness

All patients discussed how this functionality of entering and viewing blood results digitally increased timeliness of several aspects of their care. Staff had instant access to all information at the point of care, on devices that were easily portable, and this allowed fast action when needed, for example when blood test trends indicated suboptimal treatment.

### Efficiency

It was perceived that the digital system was very efficient in receiving information about their treatment from staff, for example via the patient portal or via the secure messaging function on the app, as opposed to receiving paper letters or phone calls. Texting prescription requirements also made collecting prescriptions more efficient for patients.

### Equity

The digital system supported equity of care provision and monitoring to all patients, regardless of whether they dialysed in the main hospital, a satellite unit or independently at home. Patients on home dialysis felt reassured that they would receive the same standard of monitoring as if they were in hospital, because of the direct link between the patient interface and the digital system that staff have access to, which allowed regular review of electronic versions of these records. Transplant patients also described using email and text functions for contacting their care team, not feeling disadvantaged by their self-care treatment plan any more.

### Physical health

The digital system has impacted patients’ wider lives, aside from the quality of their health care, through also improving the pillar of physical health. Examples were provided by different patients of how the patient portal was used to adjust diet according to blood test results (e.g. abnormal potassium levels), to find out whether certain foods were appropriate for their diet via the ‘menu’ function, and to continue with hobbies that involved exercise, such as paddle boarding.

### Mental health

The ability to instantly contact staff at their renal units, through the secure messaging function on the patient portal and to receive their blood results in a timely manner, supported patients to feel reassured and alleviated any anxiety, providing peace of mind and having a positive impact on their mental health.

### Social health

Participants gave examples throughout the interviews of how the patient portal helped with several aspects of their wider life and social health. This included a) their careers, by enabling them to provide list of current medications/blood test results to prove that they were within ‘normal’ ranges (i.e. that they were healthy enough to work); b) their hobbies and social lives, by helping them to inform the organisers about their condition and continue with their hobbies in a safe way, and also by enjoying meals in restaurants; c) their holidays, as they could inform others about their current medications when needed.

## Discussion

Key findings from our multi-factorial evaluation align directly with some of the national design principles to drive change and transformation, put forward by the WG in their plan for Health and Social Care [1]:

1. Prevention and early intervention: access to information on patient portal allows patients to maintain healthy lifestyles, e.g. dietary choices, to prevent worsening of their conditions or development of co-morbidities.
2. Safety: EPMA ensures that everybody involved in the care of a patient, including the patient themselves, shares the same knowledge about them and so harm is minimised.
3. Independence: EPMA, and its integration with the patient portal, supports self-care e.g. for patients on home dialysis treatments or those involved in the management of their own medicines, such as transplant anti-rejection mediation.
4. Voice: patients are informed about their condition and empowered to take an active part in managing it and can easily contact those involved in their care to express concerns they may have about their health.
5. Seamless care: access to information through EPMA/patient portal and its outputs by all healthcare services (renal and non-renal) enables seamless transition of care between them.

The findings of improved safety and seamless care by enhanced communication between the renal units and other primary, secondary or emergency care settings have been reported elsewhere [8, 30].

The idea that EPMA, particularly the patient portal linkage, had a positive impact on patient centredness and effectiveness of care was prevalent in the results of this study. Similar digital patient portals, used across the world, have had the same effect of promoting patient involvement in their own care. Preventative health behaviours, such as receiving flu vaccinations, blood pressure checks and lipid level screens were substantially higher in users of a patient portal than non-users [31]. Patient portal usage has also been associated with a significant increase in patient knowledge regarding health conditions, lower conflict in making decisions, higher self-efficacy and better collaborative relationships between patients and healthcare providers [32]. Additionally, several studies found that patient portal usage was linked with better medication adherence [33–35].

Our study did not investigate the impact of EPMA on specific health outcomes, however it was suggested in the results that physical health may be improved as a result of using functions of the patient portal. The impact of eHealth interventions on outcomes such as these in patients with chronic kidney disease was evaluated in a study by Stevenson et al. [36]. This study, a review of randomised control trials (RCTs) and quasi-RCTs, measured the impact of these interventions on dietary intake, behaviours and nutritional status among many other patient-centred and clinical parameters. Even though included studies had methodological limitations, findings showed the potential benefits that self-monitoring using electronic applications can have on dietary and fluid intake in patients.

Critical success factors during development of EPMA were the co-creation with service-users patients and staff, and the development of an in-house product, tailored to suit the needs of the service. This is in line with literature, whereby a system-wide service redesign, taking into account prescriber attitudes, patients and the existing workflow is recommended [17] and where understanding of the service specific constraints is highlighted as essential [37]. A clear implementation strategy was pivotal to national scale-up, incorporating a project initiation period, with a dedicated team of clinical staff (Delivery Team), experienced in technology. Engagement of key stakeholders at all time, with demonstrations, events, and creation of publicly available videos helped to maintain momentum and create enthusiasm about the transformation, and a suite of digitally accessible, lean and paper-light training materials supported flexible learning and multi-professional training, as suggested in literature [17]. In line with literature, staff concerns about IT skills or limitations of the system pre-implementation, including safety back-ups, were alleviated with this approach [30]. On-going monitoring and adjustment of the system post-implementation was also crucial, in line with other evidence [37].

During all phases of development, implementation and evaluation the team continued to reflect on the six core elements of the updated MRC framework for evaluating complex interventions [18], a process that informed progress throughout. This also enabled the team to anticipate and prepare for key challenges during national roll-out, including:

- Time for staff to be released for training—overcome by:
  - Lean training material in the form of short videos;
  - Real time one-to-one training in the care setting.
- Across Health Board IT barriers—overcome by a dedicated renal IT engineer to coordinate roll-out with local IT teams.
- Limited engagement from some key staff due to workload pressures—overcome by:
  - Identifying local enthusiasts and agile use of local skill mix;
  - Having a multi-professional central Delivery Team agile to fill local gaps in skill mix.

In addition, rollout during the COVID-19 pandemic meant extra service demands and periods of staff shortages. Even so, the experience and flexibility of the Delivery Team meant that implementation was completed. It is important to note that to ensure sustainability, all of these barriers remain and whilst addressing them at the point of implementation ensured this phase of the project was completed successfully, each centre needs to optimise the system’s use so it can achieve its full potential. A benefits realisation toolkit may support centres going forward and may reduce regional variation [38].

While this article has concentrated on EPMA, this is one of five elements of the Transformation Programme for *recognising*, *preventing* and *managing* kidney disease across Wales [39].

Limitations of the arm of the evaluation presented in this article include the small number of patients, which may mean that opinions may not be fully representative for all patients. This limitation was mitigated by using principles of information power to determine sample size, so that the final sample included patients receiving all of the different types of treatment (dialysis at hospital, dialysis at home and transplant recipients).

## Conclusion

EPMA was successful in improving the quality of care that people with kidney disease receive across Wales, contributed to Value-Based outcomes, and put people who deliver and access care at the heart of transformation.

## References

[1] Welsh Government. A Healthier Wales: our Plan for Health and Social care. 2018. https://gov.wales/sites/default/files/publications/2019-10/a-healthier-wales-action-plan.pdf. Accessed 07 Jun 2022

[2] Welsh Renal Clinical Network. Renal Services in Wales 2016–2020 Delivery Plan. 2016. http://www.wales.nhs.uk/sites3/Documents/773/Renal%20Disease%20Quality%20Delivery%20Plan1.pdf. Accessed 07 Jun 2022

[3] NHS. Treatment. In: Chronic kidney disease. 2019. https://www.nhs.uk/conditions/kidney-disease/treatment/. Accessed 07 Jun 2022

[4] Chief Medical Officer for Wales’s special edition annual report 2019 to 2020: protecting our health. 2021. https://gov.wales/chief-medical-officer-waless-special-edition-annual-report-2019-2020-protecting-our-health. Accessed 08 Jun 2022

[5] Shemilt K, Morecroft CW, Ford JL (2017). Inpatient prescribing systems used in NHS Acute Trusts across England: a managerial perspective. Eur J Hosp Pharm.

[6] World Health Organisation. Medication Without Harm. 2017. https://www.who.int/publications/i/item/WHO-HIS-SDS-2017.6. Accessed 07 Jun 2022

[7] Welsh Government. Transformation Fund. 2019. https://gov.wales/sites/default/files/publications/2020-09/transformation-fund-september-2020.pdf. Accessed 07 Jun 2022

[8] Franklin BD, O'Grady K, Donyai P (2007). The impact of a closed-loop electronic prescribing and administration system on prescribing errors, administration errors and staff time: a before-and-after study. Qual Saf Health Care.

[9] Nottinghamshire Healthcare NHS Foundation Trust. Outline Business Case for a Trust-wide Electronic Prescribing and Medicines Administration System. 2018. https://www.nottinghamshirehealthcare.nhs.uk/download.cfm?doc=docm93jijm4n5708.pdf. Accessed 19 Jul 2022

[10] Sheffield Teaching Hospitals NHS Trust. Outline Business Case for a Trust-wide Electronic Prescribing and Medicines Administration System. 2011. https://www.eprescribingtoolkit.com/wp-content/uploads/2020/04/sheffield-EPMA_OBC_v1.0.pdf. Accessed 19 Jul 2022

[11] Van Wilder A, Spriet I, Van Eldere J (2020). Translating data from an electronic prescribing and medicines administration system into knowledge: application to doctor-nurse time discrepancy in antibiotic ordering and administration. Med Care.

[12] Pirnejad H, Niazkhani Z, van der Sijs H (2008). Impact of a computerized physician order entry system on nurse-physician collaboration in the medication process. Int J Med Inform.

[13] Niazkhani Z, Pirnejad H, van der Sijs H (2010). Computerized provider order entry system—Does it support the inter-professional medication process? Lessons from a Dutch Academic Hospital. Method Inform Med.

[14] Westbrook JL, Li L, Georgiou A (2013). Impact of an electronic medication management system on hospital doctors’ and nurses’ work: a controlled pre-post, time and motion study. J Am Med Inform Assn.

[15] Khajouei R, Wierenga PC, Hasman A (2011). Clinicians’ satisfaction with CPOE ease of use and effect on clinicians’ workflow, efficiency and medication safety. Int J Med Informs.

[16] Jheeta S, Franklin BD (2017). The impact of a hospital electronic prescribing and medication administration system on medication administration safety: an observational study. BMC Health Serv Res.

[17] Alshahrani F, Marriott JF, Cox AR. A qualitative study of prescribing errors among multi-professional prescribers within an e-prescribing system. Int J Clin Pharm. 2021;43:884–892. 10.1007/s11096-020-01192-0 10.1007/s11096-020-01192-0 PMC8352824 33165835

[18] Skivington K, Matthews L, Simpson SA (2021). A new framework for developing and evaluating complex interventions: update of Medical Research Council guidance. Br Med J.

[19] Hart E, Bond M (1995). Action research for health and social care: a guide to practice.

[20] Cordeiro L, Soares CB. Action research in the healthcare field: a scoping review. JBI Database System Rev Implement Rep. 2018;16(4):1003–1047. 10.11124/JBISRIR-2016-003200 10.11124/JBISRIR-2016-003200 29634517

[21] Yardley L, Morrison L, Bradbury K (2015). The person-based approach to intervention development: application to digital health-related behavior change interventions. J Med Internet Res.

[22] Van den Haak MJ, De Jong MDT, Schellens PJ. Evaluation of an informational web site: three variants of the think-aloud method compared. Tech Commun. 2007;54(1):58–71. Retrieved from https://www.ingentaconnect.com/contentone/stc/tc/2007/00000054/00000001/art00006. Accessed 20 May 2022

[23] Bradbury K, Morton K, Band R (2018). Using the Person-Based Approach to optimise a digital intervention for the management of hypertension. PLoS ONE.

[24] Hawe P, Shiell A, Riley T (2009). Theorising interventions as events in systems. Am J Community Psychol.

[25] Greenhalgh T, Papoutsi C. Studying complexity in health services research: desperately seeking an overdue paradigm shift. BMC Med. 2018;16(95). 10.1186/s12916-018-1089-4 10.1186/s12916-018-1089-4 PMC6009054 29921272

[26] Tonkin-Crine S, Anthierens S, Hood K (2016). Discrepancies between qualitative and quantitative evaluation of randomised controlled trial results: achieving clarity through mixed methods triangulation. Implement Sci.

[27] World Health Organisation. Constitution of the World Health Organisation. 1948. Available from https://apps.who.int/gb/bd/PDF/bd47/EN/constitution-en.pdf. Accessed 12 May 2022

[28] Institute of Medicine (US) Committee on Quality of Health Care in America. Crossing the Quality Chasm: A New Health System for the 21^st^ Century. Washington DC: National Academies Press; 2001 25057539

[29] About PatientView—what it can do and how. http://help.patientview.org/patientview2/about/. Accessed 14 Jun 2022

[30] Mills PR, Weidmann AE, Stewart D. Hospital staff views of prescribing and discharge communication before and after electronic prescribing system implementation. Int J Clin Pharm. 2017;39**:**1320–1330. Doi : 10.1007/s11096-017-0543-2 10.1007/s11096-017-0543-2 PMC5694510 29076013

[31] Huang J, Chen Y, Landis JR (2019). Difference between users and nonusers of a patient portal in health behaviors and outcomes: retrospective cohort study. J Med Internet Res.

[32] Hae-Ra H, Gleason K, Chun-An S (2019). Using patient portals to improve patient outcomes: systematic review. JMIR Hum Factors.

[33] Saberi P, Catz SL, Leyden WA (2015). Antiretroviral therapy adherence and use of an electronic shared medical record among people living with HIV.

[34] Delblanco T, Walker J, Bell SK (2012). Inviting patients to read their doctors’ notes: a quasi-experimental study and a look ahead. Ann Intern Med.

[35] Ammenwerth E, Schenll-Inderst P, Hoerbst A (2012). The impact of electronic patient portals on patient care: a systematic review of controlled trials. J Med Internet Res.

[36] Stevenson JK, Campbell ZC, Webster AC, et al. eHealth interventions for people with chronic kidney disease. Cochrane Database Syst Rev. 2019; 2019(8): CD012379. 10.1002/14651858.CD012379.pub2 10.1002/14651858.CD012379.pub2 PMC6699665 31425608

[37] Seidling HM, Stützle M, Hoppe-Tichy T, et al. Best practice strategies to safeguard drug prescribing and drug administration: an anthology of expert views and opinions. Int J Clin Pharm. 2016;38**:**362–373. Doi: 10.1007/s11096-016-0253-1 10.1007/s11096-016-0253-1 26964781

[38] Achieving benefits realisation. Medicines Optimisation Network. The University of Edinburgh. 2022. https://www.eprescribingtoolkit.com/achieving-benefits-realisation/ Accessed 19 Jul 2022

[39] Welsh Government Transformation Fund 2018–20—Guidance. 2019. https://gov.wales/sites/default/files/publications/2019-03/welsh-government-transformation-fund-2018-20-guidance.pdf. Accessed 08 Jun 2022

